# Human Genome-Wide RNAi Screen for Host Factors That Facilitate *Salmonella* Invasion Reveals a Role for Potassium Secretion in Promoting Internalization

**DOI:** 10.1371/journal.pone.0166916

**Published:** 2016-11-23

**Authors:** Joshua M. Thornbrough, Adarsh Gopinath, Tom Hundley, Micah J. Worley

**Affiliations:** 1 Department of Biology, University of Louisville, Louisville, KY, 40292, United States of America; 2 Department of Microbiology and Immunology, University of Louisville, Louisville, KY, 40202, United States of America; Universitat Osnabruck, GERMANY

## Abstract

*Salmonella enterica* can actively invade the gastro-intestinal epithelium. This frequently leads to diarrheal disease, and also gives the pathogen access to phagocytes that can serve as vehicles for dissemination into deeper tissue. The ability to invade host cells is also important in maintaining the carrier state. While much is known about the bacterial factors that promote invasion, relatively little is known about the host factors involved. To gain insight into how *Salmonella enterica* serovar Typhimurium is able to invade normally non-phagocytic cells, we undertook a global RNAi screen with *S*. Typhimurium-infected human epithelial cells. In all, we identified 633 genes as contributing to bacterial internalization. These genes fall into a diverse group of functional categories revealing that cytoskeletal regulators are not the only factors that modulate invasion. In fact, potassium ion transport was the most enriched molecular function category in our screen, reinforcing a link between potassium and internalization. In addition to providing new insights into the molecular mechanisms underlying the ability of pathogens to invade host cells, all 633 host factors identified are candidates for new anti-microbial targets for treating *Salmonella* infections, and may be useful in curtailing infections with other pathogens as well.

## Introduction

*S*. Typhimurium produces diarrheal disease in humans. It is generally self-limiting but can be serious in infants and the elderly and also in immunocompromised patients. The closely related *Salmonella enterica* serovar Typhi on the other hand causes typhoid fever, a more serious, systemic disease, which is potentially fatal in otherwise healthy people. The public health threat is compounded by the emergence of multi-drug resistant strains[1]. In fact, strains of *S*. Typhi resistant to ciprofloxacin and most other antibiotics have become endemic in India and have also been reported in developed countries[2]. *Salmonella* is also studied because it is a convenient model organism for dissecting basic pathogenic properties such as the ability of microorganisms to invade host cells.

Following consumption, *Salmonella* deploys two distinct type III secretion systems harbored by *Salmonella* pathogenicity islands 1 and 2 (SPI-1 and SPI-2)[3]. These systems span the bacterial envelope and either the plasma or vacuolar membranes of host cells[3]. They function as ‘molecular syringes’, injecting a cocktail of effectors into host cell cytosol that manipulate various cellular functions in ways that benefit the pathogen[3]. *Salmonella* utilizes SPI-1 to invade cells and invoke an inflammatory response[4–6], important components of the gastrointestinal (GI) phase of disease. The role of SPI-1 in *Salmonella* pathogenesis in not limited to the GI phase of disease however, as numerous genes located in SPI-1 are synthesized at late stages of infection in the murine model of acute typhoid fever and are also required for persistence and shedding in a murine model of the carrier state[7–9]. After invasion of host cells, *Salmonella* utilizes SPI-2 to grow within them[10–12] and to influence phagocyte movement[13–15]. Inside cells, *Salmonella* resides within the *Salmonella* containing vacuole (SCV), which diverges from the endocytic pathway due to the action of both SPI-1 and SPI-2[16].

The host processes that are required for bacterial entry and the early establishment of a successful infection are not yet fully elucidated. With a global RNAi screen, we identified 633 human genes, which facilitate *S*. Typhimurium invasion of MCF-7 cells, including a large suite of genes involved in potassium transport. This study should contribute to the study of both GI and systemic salmonellosis and the genes identified may be able to serve as new anti-microbial targets.

## Results

### Human epithelial cell screen to identify host factors required for *S*. Typhimurium invasion

In our genome-wide screen, using a reverse transfection protocol, we targeted ≈22,000 known or predicted genes in human epithelial-like MCF-7 cells with siRNAs arrayed in 384 well plates. Four independent siRNAs, two in one plate and another two in a parallel plate targeted each gene. This 2 X 2 format reduced the likelihood that off-target effects accounted for the phenotypes. We incubated the cells for 72 hours following transfection, before infecting them with *S*. Typhimurium for thirty minutes. The 72-hour incubation allowed even long-lived proteins a chance to turn over. The strain expressed a copy of the GFP from a plasmid. Gentamicin was used to eliminate extracellular bacteria. Eighteen hours after infection, we fixed the cells, stained nuclei with Hoechst 33342 and determined the percentage of host cells that were infected by measuring GFP fluorescence within the estimated periphery of cells. The 18 hour incubation allowed us to identify host genes that modulated the intracellular growth of the bacteria, which were presented elsewhere[17], as well as those that facilitate invasion. Genes that reduced bacterial infection and had a p-value ≤ 0.02 in parallel wells were considered hits. We did not consider as hits wells that had fewer than 500 cells present as there was a positive correlation between the percentage of cells infected and the total present below this number, but not above it (S1 Fig). The phenotypes of some of the hits may be attributable to physiology changes that allowed gentamicin to build up inside of the epithelial cells. However, this is probably not the case for the majority of the hits as a cell affected in this manner would likely be unhealthy and moreover we did not identify genes involved in drug efflux as would be expected if it was an issue.

### The host network that promotes *S*. Typhimurium invasion of epithelial cells

**Fig 1:**
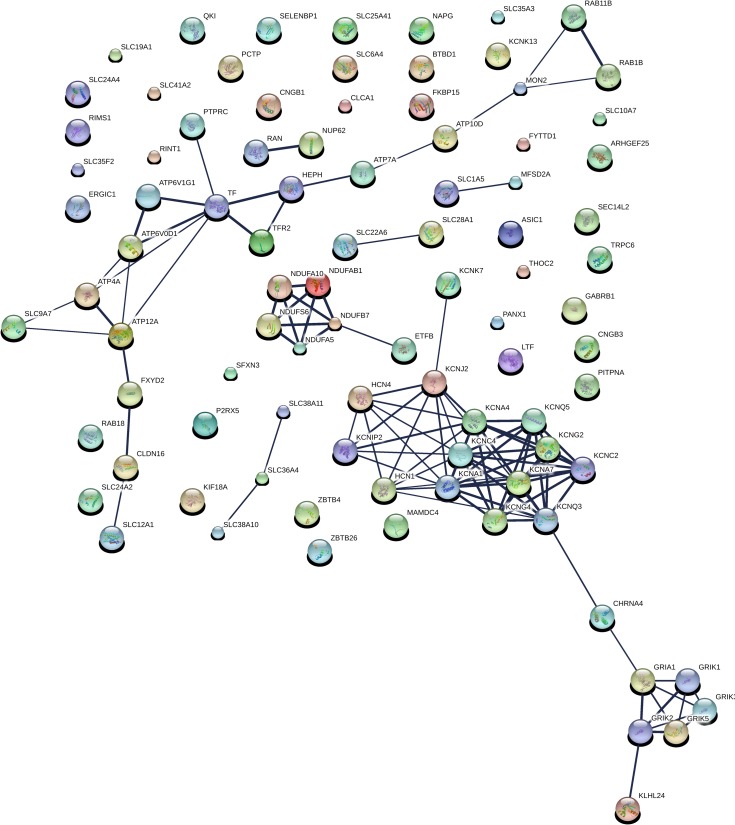
The most prominent Sting-derived subnetwork that promotes the invasion of epithelial cells by *S*. typhimurium. The subnetwork is involved in potassium transport.

**Fig 2:**
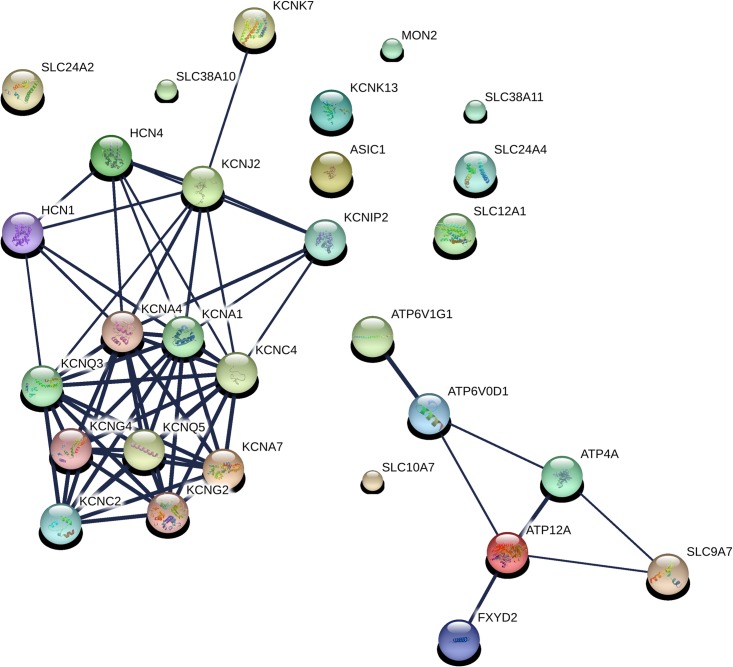
The second most prominent Sting-derived subnetwork that promotes the invasion of epithelial cells by *S*. typhimurium is also involved in potassium transport.

**Fig 3:**
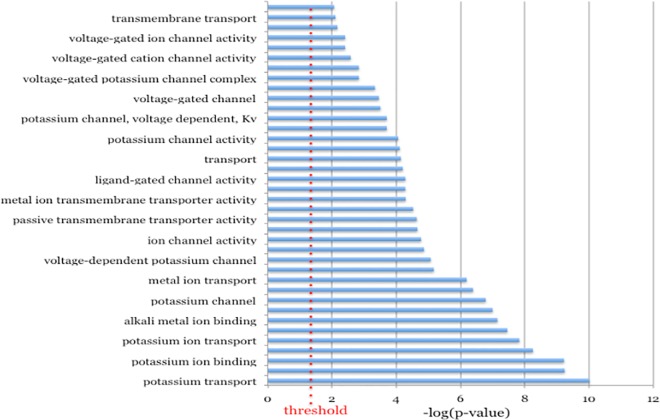
The overrepresented molecular function categories for the hit list. The–log(p-value) is plotted with 0.05 being the significance threshold.

In all, 633 genes were identified as facilitating *S*. Typhimurium entry into MCF-7 cells. These genes are described in S1 Table. Information on all of the genes screened can be found in S2 Table. The two most prominent string-derived subnetworks are displayed in Figs 1 and 2. Both subnetworks are involved in potassium ion transport. The members of these subnetworks are described in S3 and S4 Tables. The molecular function categories of 633 genes, for which there was significant enrichment are show in Fig 3. The most enriched category by far, with a remarkable p-value of 9.86 X 10^−11^ was potassium transport. Potassium levels, but not potassium transport, have been shown to play a role in *Salmonella* pathogenesis. The bacterial potassium transporter Trk and external potassium modulate *Salmonella* type III secretion, invasion of epithelial cells and pathogenesis in mice and chickens[18,19]. In addition to genes involved in potassium transport, the molecular function categories of metal ion binding and transport were also highly enriched with p-values of 7.15 X 10^−8^ and 6.49 X 10^−7^ respectively. Iron has been shown to play a role in regulating invasion[20].

### Comparison with another RNAi screen that identified genes involved in invasion

A different group performed an RNAi screen on the ‘druggable’ genome with HeLa epithelial-like cells and *S*. Typhimurium to identify genes involved in invasion. The genes shared between our study (633) and Misselwitz et. al.[21] (90) are CYP26C1, TUBA4B, CYP27A1 and MUC5AC. Cytochrome P450 26C1 (CYP26C1) is inferred from electronic annotation to play a role in retinoic acid metabolism. Although it was identified in both studies, it is not clear how it would contribute to invasion. Putative tubulin-like protein alpha-4B (TUBA4B) is electronically inferred to bind GTP and to be a structural component of the cytoskeleton and accordingly, could plausibly play a role in internalization. Sterol 26-hydroxylase, mitochondrial (CYP27A1) is electronically inferred to catalyze the first step in sterol intermediate side chain oxidation. It is unclear how this would promote invasion. Mucin-5AC (MUC5AC) is an extracellular matrix structural constituent, which is a gel forming glycoprotein of gastric and respiratory tract epithelial that binds microorganisms that are then removed by the mucocilary system thereby protecting the epithelial cells from infection[22]. It seems possible that this gene was identified in both screens because the encoded protein facilitates close association of the epithelial cells and *S*. Typhimurium without a mucocilary system in the *in vitro* systems to remove the bacteria.

### Identification of genes that likely cause the SCV to fuse with lysosomes

We previously published the human genes we identified as promoting the growth of *S*. Typhimurium within MCF-7 cells[17] This list of 252 genes had 43 in common with the ones presented here that promote invasion. The absence of the genes likely does not affect invasion but rather causes the SCV to merge with the lysosome. The genes that fall in both groups are shown in S5 Table. Notable among this list are copine 5 (CPNE5), Ras-related protein 1B (Rab1b), Myotubularin-related protein 3 (MTMR3) and the lysosomal H^+^ transporting subunit V0D1 (ATP6V0D1). CPNE5 functions in membrane trafficking and its absence could affect the fate of the early SCV[23] as could Rab1b, as Rabs are small GTPases that are key regulators of intracellular membrane trafficking[24]. MTMR3 is a phosphatase that acts on phosphatidyl inositol (PtdIns) species that control the endocytic pathway[25]. ATP6V0D1 is a component of the vacuolar ATPase that is responsible for acidifying the SCV[26]. *S*. Typhimurium uses acid as a cue to express SPI-2 related virulence genes which are necessary for avoiding fusion of the SCV with lysosomes[27].

### The identification of human genes that could participate in *S*. Typhimurium entry into human cells

**Fig 4:**
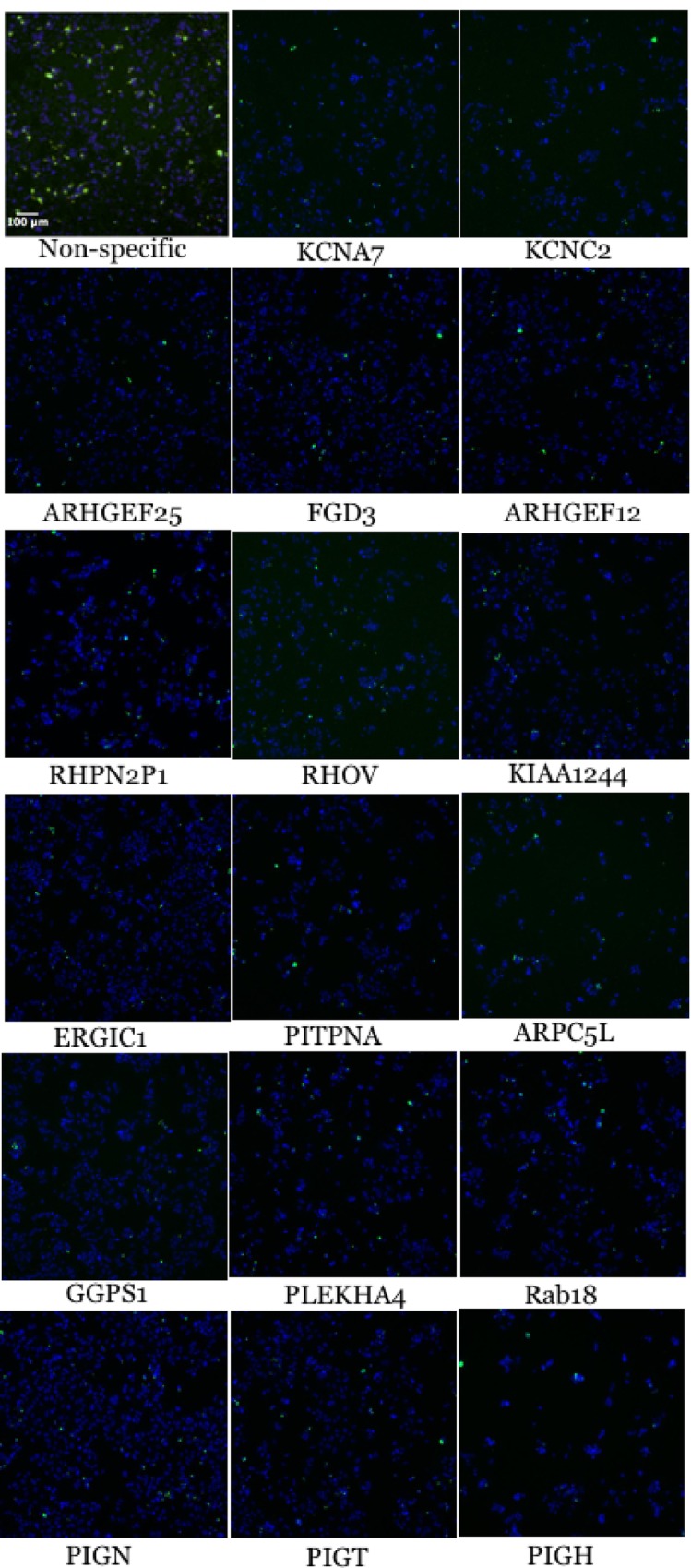
The microscopy data from some of the hits that can immediately be rationalized as playing a role in the invasion of epithelial cells by *S*. typhimurium. Host cell nuclei are in blue and the bacteria are in green. The upper left image is a representative non-silencing control.

While many of the 633 host factors identified as mediating the internalization of *S*. Typhimurium likely play novel roles in the process and fall into a diverse group of functional categories, numerous hits can be immediately rationalized as proteins that could facilitate invasion. Some of these hits are described below. The raw microscopy data for these hits is displayed in Fig 4.

KCNA7 is predicted to be a potassium voltage-gated channel of the shaker-related subfamily. KCNC2 is a potassium voltage-gated channel of subfamily C. In response to the voltage difference across the membrane, KCNC2 assumes an open or closed conformation, which when open forms a potassium selective channel that potassium ions can pass through[28]. KCNA7 and KCNC2 are two among at least 22 genes identified that play roles in mediating potassium transport. Potassium ions are known to activate SPI-1 and invasion[1,19].

We identified three Rho guanine nucleotide exchange factors (GEFs), one phophilin-2 like protein, which binds Rho proteins as well as one actual member of the Rho family as contributing to the invasion of epithelial cells by *S*. Typhimurium. Rho GEF 25 (ARHGEF25) and FYVE, RhoGEF and PH domain-containing protein 3 (FGD3) as well as Rho GEF 12 (ARHGEF12) are hits that can be easily rationalized as playing roles in the invasion of epithelial cells by *Salmonella* even though they have not been previously implicated in this process[29]. These proteins activate Rac, Cdc42 and Rho that are all well established to play a role in microbial internalization. *Salmonella* secretes the SPI-1 type III effectors SopE and SopE2 into epithelial cells, where they serve as nucleotide exchange factors (GEF) for the small GTP binding proteins Rac and Cdc42, catalyzing the exchange of GTP for GDP[30,31]. These proteins are active when bound to GTP and control downstream molecules that trigger the formation of highly branched and interconnected actin networks that lead to the formation of membrane ruffles, which facilitate *Salmonella* entry. While bacterially encoded GEFs have been known to facilitate entry for some time, this is to the best of our knowledge the first report of endogenous GEFs being required. It will be interesting to determine if these GEFs are directly targeted by *Salmonella* secreted proteins. We also identified putative rhophilin-2-like protein (RHPN2P1) as contributing to *S*. Typhimurium invasion, which is a member of the rhophilin family of Rho binding proteins. The protein binds both GTP- and GDP-bound RhoA and GTP-bound RhoB and may be involved in the organization of the actin cytoskeleton[32]. In addition to Rho GEFs and RHPN2P1, we also identified a Rho family member, RhoV, which may stimulate the JNK pathway to influence the actin cytoskeleton[33].

ARFGEF3 has Arf GTPase activity, which could plausibly stimulate *Salmonella* invasion of epithelial cells as it could trigger the activation of the WAVE regulatory complex, which *Salmonella* recruits to the underlying membrane where it governs actin polymerization. In fact, invading *Salmonella* are known to recruit and activate Arf1 to facilitate ruffling and Arf3 and Arf6 also promote invasion[34]. Thus, ARFGEF3 could be a new player in mediating the internalization of *Salmonella* by human cells.

The identification of ER-Golgi intermediate compartment-1 (ERGIC1) as one of the stronger hits in our screen was at first surprising. This gene encodes an ERGIC cycling membrane protein that regulates the stability of other members of this protein family. Recently, it was reported that depletion of the COPI complex resulted in a strong invasion defect for *S*. Typhimurium[21]. Like ERGIC1, the COPI complex regulates traffic between the ER and the Golgi apparatus. Depleting cells of COPI resulted in the mislocalization of cholesterol, sphingolipids, Rac1 and Cdc42 away from the plasma membrane into a large intracellular compartment, which decreased virulence factor injection and membrane ruffling[21].

ERGIC1 is a novel cycling membrane protein, which shares homology with two yeast proteins that are enriched in COPI vesicles: Erv41p and Erv46p. ERGIC1 localizes to the ERGIC and colocalizes with the human homologs of Erv41p and Erv46p. Covalent cross-linking revealed that ERGIC1 interacts with human ERGIC3 (hErv46). Depleting cells of ERGIC1 increases the turnover rate of ERGIC3 however the converse does not occur. Silencing ERGIC2 increases the turnover of ERGIC3, but not ERGIC1. It was thus proposed that ERGIC1 serves as a modulator of the ERGIC2-ERGIC3 complex through the stabilization of ERGIC3[35]. The function of these three proteins in mammalian cells remains to be fully elucidated. Depletion of ERGIC1 and ERGIC3 does not effect protein or glycoprotein secretion. Since ERGIC3 and ERGIC2 are interdependent, this double knock down is actually a triple knock down. This data suggests that these proteins are not essential for vesicular trafficking. The invasion defect in ERGIC1 depleted cells may be due to the mislocalization of cholesterol away from the plasma membrane as cholesterol in the plasma membrane is required for *S*. Typhimurium invasion[21]. Interestingly, in our screen, depleting cells of components of the COPI complex adversely affected host cell health whereas reducing the level of ERGIC1 did not (S2 Fig).

PtdIns transfer protein (PITPNA) can transfer phospholipids between membranes *in vitro*[36]. PtdIns species are known to play roles in bacterial infection[37]. They can provide a negative charge to the inner side of the plasma membrane that attracts and anchors signaling and effector molecules that trigger phagosome formation. The loss of charge following their metabolism as phagosomes seal then causes the dissociation of proteins with the concomitant termination of signaling and cytoskeleton assembly[38].

By sequence similarity, Actin-related protein 2/3 complex subunit 5-like protein (ARPC5L) is predicted to function as a component of the Arp2/3 complex. This complex is involved in regulation of actin polymerization and together with an activating nucleation-promoting factor mediates the formation of branched actin networks. It is required for the invasion of epithelial cells by *Salmonella*[39].

Interfering with geranylgeranyl diphosphate synthase 1 (GGPS1) was also determined to significantly reduce *S*. Typhimurium invasion. This enzyme synthesizes geranylgeranyl diphosphate, which is an important precursor to geranylated proteins, from farnesyl diphosphate and isopentenyl diphosphate[40]. Another study showed that treating cells with geranylgeranyltransferase inhibitor, prevented Rho GTPases from being geranylgeranylated, dislocalizing them from the plasmid membrane thereby inhibiting the invasion of HeLa cells by *S*. Typhimurium[21], likely explaining why it came up in our screen.

Pleckstrin homology domain containing family A (phosphoinositide binding specific) member 4 (PLEKHA4) was another invasion hit. It binds specifically to phosphatidylinositol 3-phosphate (PtdIns3P) but not other PtdIns species, although the consequence of this interaction is not known[41]. It could plausibly play a role in mediating *S*. Typhimurium invasion, as PtdIns3P has been implicated in the process and can be found at the ruffling membrane and nascent phagosomes[42].

Rabs are known to regulate the endocytic pathway and several of them have been implicated in SCV biogenesis and development and been shown to be modulated by *S*. Typhimurium[43]. Rab18, one of our invasion hits, is not known to be involved in *Salmonella* pathogenesis, but it has been implicated in facilitating transport between the plasma membrane and early endosomes and in light of our results, likely plays a role[43].

GPI ethanolamine phosphate transferase 1 (PIGN), phosphatidylinositol glycan anchor biosynthesis, class T (PIGT) and phosphatidylinositol glycan anchor biosynthesis, class H (PIGH) are all three involved in attaching glycosylphosphatidyl inositol (GPI) anchors to proteins and localizing them to the apical side of the epithelial cell plasma membrane[44]. Here, these proteins could interact with microbes and facilitate invasion.

### Confirmation of the role of potassium secretion in promoting invasion

**Fig 5:**
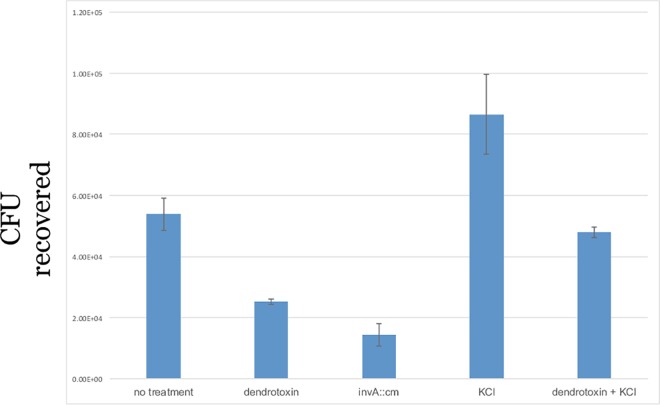
An invasion assay was performed with MCF-7 cells that were untreated or treated with dendrotoxin or treated with dendrotoxin plus potassium or just potassium. An *invA*::*cm* strain is included as a control. The experiment was performed in duplicate on two independent occasions. Error bars depict the standard error of the mean. *p-value <0.05

To confirm the role of potassium secretion in promoting the invasion of epithelial cells by *S*. Typhimurium, we utilized alpha dendrotoxin, a chemical inhibitor of a subset of voltage-gated potassium channels. In this experiment, cells were either untreated or treated with dendrotoxin or treated with dendrotoxin and potassium and infected with bacteria. We observed that dendrotoxin deters the invasion of epithelial cells by *S*. Typhimurium and that addition of exogenous potassium to the media alleviates the inhibition (Fig 5).

## Discussion

While many microbial factors that *S*. Typhimurium uses to invade normally non-phagocytic cells have been identified, relatively little is known about the host factors involved in this process. This genome-wide screen identifies the human genes exploited by *S*. Typhimurium and likely other pathogens as well to facilitate microbial entry into epithelial cells. In addition to generating much new information that will ultimately help unravel the molecular mechanisms underlying the ability of *S*. Typhimurium to invade such human cells, all of the 633 factors identified here are candidate for a new class of anti-microbial targets.

The set of genes identified in our screen had very modest overlap with an RNAi screen designed to detect host factors associated with invasion mediated by the SPI-1 type III effector SopE. There are many reasons for the lack of significant overlap. The study by Misselwitz et. al utilized a different *S*. Typhimurium strain. They used strain SL1344 whereas we employed strain 14028s. Also, their strain lacked three effectors that can affect invasion, SipA, SopE2 and SopB whereas we used wild type 14028s. Their screen was SopE driven whereas strain 14028s lacks SopE. Additionally, a different siRNA library was used, which was only composed of the 6,978 ‘druggable’ genes whereas we screened the entire genome of apoproximately 22,000 genes. Further, different host cells were used. We employed MCF-7 cells whereas Misselwitz et. al used HeLa cells. Finally, we did not include as hits genes whose absence affected host cell health.

The most enriched molecular function category by far was potassium transport. There is precedence for the manipulation of host ion transport by *Salmonella*. *Salmonella* injects the SPI-1 type III effector SopB into epithelial cells to manipulate PtdIns-P species in order to among other things, produce PtnIns (1,4,5,6)P_4_ to trigger chloride secretion and concomitant fluid entry into the GI tract, resulting in diarrhea[45]. The effect is due to the antagonism of ion channel closing. To the best of our knowledge, nobody has tested whether or not *Salmonella* stimulates the secretion of potassium as well as chloride. In light of our results, it seems likely that it may stimulate potassium secretion as external potassium is used as a cue by the bacteria to up-regulate SPI-1 and thereby promote invasion[18,19]. While our results suggest that potassium secretion by epithelial cells is a requirement for efficient *Salmonella* invasion, additional work will be required to determine if epithelial cells continuously secrete potassium, which the bacteria use as a cue to invade or it is a direct or indirect result of the presence of the bacteria.

Another study demonstrated that the CopI complex, the components of which have their stability regulated by ERGIC1, plays a role in regulating plasma membrane levels of cholesterol[21]. Caveolae are long-lived membrane microdomains composed of caveolins, cavins and a cholesterol rich membrane. These cholesterol-enriched domains can bind activated Rho GTPases via their geranylgeranyl anchors[46,47]. Misselwitz et. al. showed that Rho GTPases, which are responsible for host cell ruffling and concomitant *S*. Typhimurium entry are not localized to the plasma membrane in cells depleted for components of the CopI complex[21]. We observed that depleting cells of components of the CopI complex dramatically affected the health of the host cells whereas silencing ERGIC1 did not. Perhaps silencing ERGIC1 results in only a partial loss of CopI complex components that affect *S*. Typhimurium invasion but still allow for basic host cells processes to be carried out. The ability of ERGIC1 to increase the turnover rate of numerous ERGIC proteins, coupled with its apparent dispensability for the short-term health of host cells, may make it a unique anti-microbial target for a variety of pathogens as the requirement for cholesterol-enriched microdomains in the plasma membrane is not unique to *S*. Typhimurium. In fact, these internalization requirements are shared amongst *Staphylococcus aureus*[48,49], *Escherichia coli*[48,50,51], *Listeria monocytogenes*[52–55], *Mycobacterium fortuitum*[50], *Mycobacterium tuberculosis*[56], *Chlamydia caviae*[57], *Chlamydia trachomatis*[58,59], influenza virus[60,61], *Shigella flexneri*[62], *Brucella* abortus[63], hepatitis C virus[64,65] and vesicular stomatitis virus[21].

MTMR3, a protein whose absence likely results in the SCV merging with the lysosome is also interesting to consider further. The action of MTMR3 may change the electrostatic charge on the SCV causing Rabs that promote the fusion of endosomes with lysosomes to disassociate. The type III effector SopB, in addition to generating PtnIns(1,4,5,6)P_4_ was speculated to recruit Rab5 vacuoles in part by dephosphorylating PtdIns(4,5)P_2_, a tetravalent anion at physiological pH, thereby reducing the negative charge on the SCV. This may stimulate the association of some Rabs, but has been shown by Bakowski et. al. to cause other Rabs such as 8b, 13, 23 and 35, which share a polycationic prenyl membrane targeting motif in their carboxyl terminus to dissociate[66]. This dissociation halts the endocytic pathway. Smith et. al. in fact demonstrated that Rabs 23 and 35, which disassociate when SopB alters the electrostatic charge of the SCV, normally promote phagosome-lysosome fusion[43]. This likely causes the observed delay in SCV development where Rab7 containing SCVs do not fuse with late endosomes. This is reminiscent of the “pregnant pause” mechanism used by *Legionella pneumophila* and other intracellular bacterial pathogens, which temporarily halt maturation of their vacuole so they can form a replicative niche before the vacuole matures to the next state[67]. However, importantly, another study demonstrated that after a while, the PtdIns(3)P 5 kinase PIKfyve phosphorylates PtdIns(3) to PtdIns(3,5)P_2_[27], which should increase the negative charge on the SCV, triggering the reassociation of Rabs 8b, 13, 23 and 35 thereby restarting the endocytic pathway. Whether it is host or the microbe that does this is not known. It may be the bacteria, as *Salmonella* cannot replicate in this intermediate SCV as acidification of the vacuole subsequent to the pregnant pause is necessary for activating a subset of intracellular virulence gene including those associated with SPI-2[27]. What then prevents the SCV from fusing with the lysosome when Rabs 23 and 35 re-associate is an open question that nobody has considered. *Salmonella* may recruit MTMR3 to the SCV at this point to prevent fusion with the lysosome, through its ability to dephosphorylate PtdIns(3,5)P_2_, which should decrease the negative charge on the SCV that was increased by PIKfyve, causing Rabs 8b, 13, 23 and 35 to disassociate for a second time. PLEKHA4 could also be involved in SCV biogenesis as it is known to bind PtdIns3P[41]. While the consequences of this interaction are not known, it is possible that it facilitates phosphorylation to PtdIns(3,5)P_2_, countering the effect of SopB, which is necessary for *Salmonella* virulence.

It is also interesting to consider the role of Rab1b in *Salmonella* pathogenesis. Rab1b is involved in the secretory pathway between the ER and the Golgi and is required for autophagosome formation[68]. There is conflicting evidence in the literature about why we identified it. One study showed that some *Salmonella* escape the SCV and grow in the cytosol where they are targeted by autophagy and their growth is reduced[69]. Another more recent study demonstrated that autophagy facilitates *Salmonella* replication in HeLa cells[70]. In fact, knockdown of Rab1, which is required for autophagy, inhibits *Salmonella* replication[71]. It is possible that Rab1b depletion prevented *Salmonella* from growing at all inside host cells and would be more accurately described as having an intracellular growth defect than an invasion defect.

With a genome-wide RNAi-based forward genetic screen, we have identified 633 host factors that are involved in the internalization of *S*. Typhimurium by human epithelial cells. These factors fall into a group of diverse functional categories, indicating that invasion involves much more than merely cytoskeletal regulators. The most notable category was genes involved in potassium transport. In future work, it will be important to determine which if any *Salmonella* virulence factors directly stimulate the secretion of potassium ions. It will also be important to confirm and characterize some of the host factors that facilitate invasion in detail. The genome-wide results from our human RNAi screen provide us with new insights into how *Salmonella* actively invades human cells.

## Materials and Methods

### Bacterial and eukaryotic cell growth conditions

As previously described[17], *S*. Typhimurium 14028s expressing the GFP from pACYC184 was grown in Luria Bertani broth (LB; Sigma-Aldrich, St. Louis, MO), supplemented with 10μg/mL tetracycline for 18 hrs at 37°C with aeration. The GFP was from *Pontellina plumata*. After 18 hours of growth, the bacteria were diluted 1:33 in fresh LB and subcultured for 3.5 hrs at 37°C with agitation to induce SPI-1 expression and then used to infect MCF-7 cells (ATCC, Manassas, VA). MCF-7 cells were cultured in Iscove’s Modified Dulbecco’s Medium (IMDM; Invitrogen, Carlsbad, CA) supplemented with 7.5% fetal bovine serum (FBS; Sigma-Aldrich) without antibiotics. Cell culture was maintained at 37°C in 5% CO_2_ in a humidified tissue culture incubator. The cells were subcultured 8.00 x 10^4^ cells/cm^2^ every fourth day.

### Infections and Gentamicin protection assay

These assays were performed as previously described[17]. Briefly, MCF-7 cells were infected with *S*. Typhimurium at an MOI of 100. An MOI of 100 was chosen because we empirically determined by testing a range of cell densities and MOIs that 3,000 host cells and an MOI of 100 resulted in the largest intracellular growth phenotype. This was important for identifying genes that normally promote intracellular growth, which were reported elsewhere[17]. In follow up experiments to confirm the role of potassium secretion in promoting invasion we used an MOI of 25. Following infection, the cells were then centrifuged at 230 rcf for 10 min at room temperature. The cells were then incubated at 37°C in 5% CO_2_ for 30 min so that the bacteria could invade. The infected cell culture was then washed twice with PBS. After washing, the cells were incubated in IMDM supplemented with gentamicin (Gibco, Carlsbad, CA) at a concentration of 100 ug/mL for one hour at 37°C in 5% CO_2_ to kill extracellular bacteria. The cells were washed two times with PBS after the gentamicin kill and then incubated in IMDM supplemented with gentamicin at a concentration of 10 ug/mL at 37°C in 5% CO_2_ for 18 hours before imaging.

### High-throughput siRNA screen of human genome

As previously described[17], a siRNA library was obtained from the Duke University RNAi screening facility targeting the human genome (Human whole-genome siRNA library v1.0; Qiagen, Valencia, CA). The library came in a 2 X 2, parallel plate format with four individual siRNAs targeting each gene arrayed in two sets, AB and CD. MCF-7 cells were seeded in 384 well clear flat bottom plates (Corning 3712; Corning, Lowell, MA) at a density of 3000 cells per well. Reverse transfections were carried out with 0.05uL Dharmafect 4 (Dharmacon, Lafayette, CO) and 20 nM siRNA/well in a total volume of 50uL. Transfection efficiency was optimized utilizing AllStars Hs Cell Death Control siRNA (Qiagen), a blend of siRNAs that are toxic and counting how many cells died. Efficiency was determined to be > 95% under these conditions. 72 hours after transfection, MCF-7 cells were infected with bacteria induced for SPI-1 expression and fixed eighteen hours later with 6% paraformaldehyde (Sigma-Aldrich) in 1 X PBS (Invitrogen) for 1 hour. Subsequently, the cells were washed two times with PBS. Nuclei were stained with Hoechst 33342 (Sigma-Aldrich) and automated imaging and analysis was performed on each well with the Cellomics ArrayScan VTI High Content imaging system (Thermo Scientific, Pittsburgh, PA). Images were analyzed via trained algorithm that first identified nuclei stained with Hoechst 33342. Next, the estimated cellular margins were established by extending the nuclear margin a predetermined radial distance. Only bacteria within this region were analyzed to avoid including any residual extracellular bacteria. Invasion defects were identified utilizing a Wilcoxin sum rank method for the percentage of infected cells in each well. Genes that reduced bacterial infection and had a p-value ≤ 0.02 in parallel wells were selected for follow-up. We normalized using Z-score based on mean fluorescence of plate/day batch. A p-value of 0.02 was chosen arbitrarily to allow for both enrichment and a manageable number of hits to follow-up.

### Bioinformatics

Genes meeting selection criteria were manually curated with the National Center for Biotechnology Information (NCBI; http://ncbi.nlm.nih.gov) gene database. Functional clusters were analyzed with the Database for Annotation, Visualization and Integrated Discovery (DAVID) v6.7 (http://david.abcc.ncifcrf.gov/). Parameters were set to default with the following exceptions, EASE score was set for 0.05 and only clusters with an enrichment score ≥ 1.0 were selected for further analysis. For developing networks and assessing potential protein interactions the Search Tool for the Retrieval of Interacting Genes/Proteins (STRING) v9.1 (http://string-db.org/) and assessed for significant interaction enrichment within the whole genome and druggable genome categories.

### Confirmation of the role of potassium secretion in promoting invasion

MCF-7 cells were untreated or treated with 10μM alpha dendrotoxin (VWR). Twenty-four hours later, the cells were infected with *S*. Typhimurium grown under SPI-1 inducing conditions as described above at an MOI of 25. To one group of wells, KCl was added to a final concentration of 3.8mM. Bacteria were centrifuged onto the monolayers at 230g for ten minutes and then incubated for thirty minutes. After the thirty minute invasion, the infected cells were washed three times with PBS and overlaid with fresh media supplemented with 100μg/mL gentamicin and incubated for one hour. After the gentamicin kill, the infected cells were washed three times with PBS and lysed in 1% Triton X-100 and CFU recovered on agar plates

## Supporting Information

S1 Fig The percentage of cells infected for each well was plotted against the number of cells present. We empirically determined that there was no longer a relationship between the two beyond 500 cells per well.
(TIF)

S2 Fig Depleting cells of members of the CopI complex but not ERGIC1 adversely affects the health of host cells. (TIF)

S1 Table The list of 633 hits and their annotations. (XLSX)

S2 Table All gene screened and their annotations. (XLSX)

S3 Table The list of genes in the first subnetwork. (XLSX)

S4 Table The list of genes in the second subnetwork. (XLSX)

S5 Table Genes that were identified as promoting growth and invasion whose absence likely cause the SCV to fuse with the lysosome. (XLSX)
