# Supplementary figures and images for: Human Genome-Wide RNAi Screen for Host Factors That Facilitate Salmonella Invasion Reveals a Role for Potassium Secretion in Promoting Internalization
Source: PLoS One. 2016 Nov 23;11(11):e0166916. doi: 10.1371/journal.pone.0166916 (PMC5120809; doi:10.1371/journal.pone.0166916)

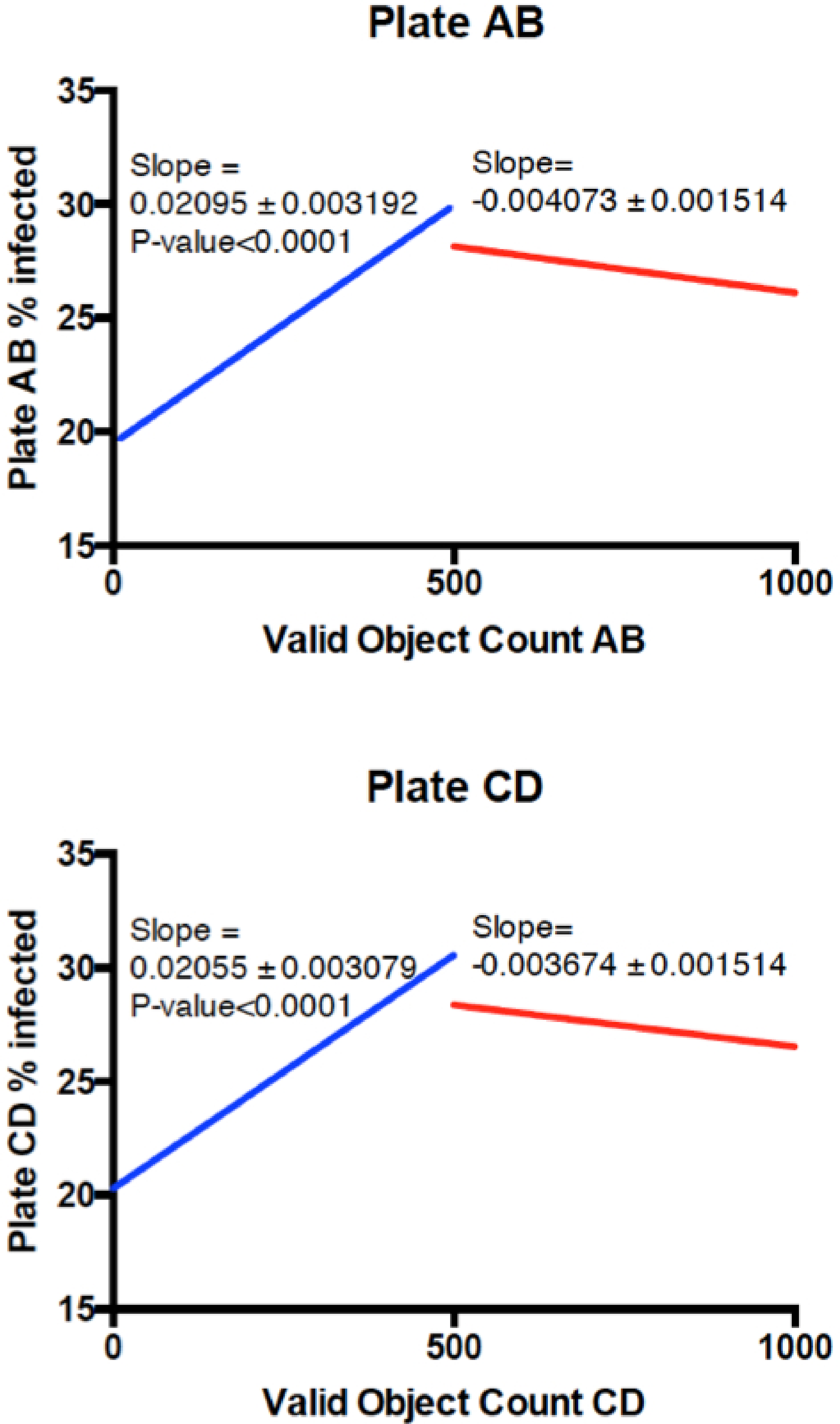

Supplement: S1 Fig — We empirically determined that there was no longer a relationship between the two beyond 500 cells per well. (TIF) [file pone.0166916.s001.tif]

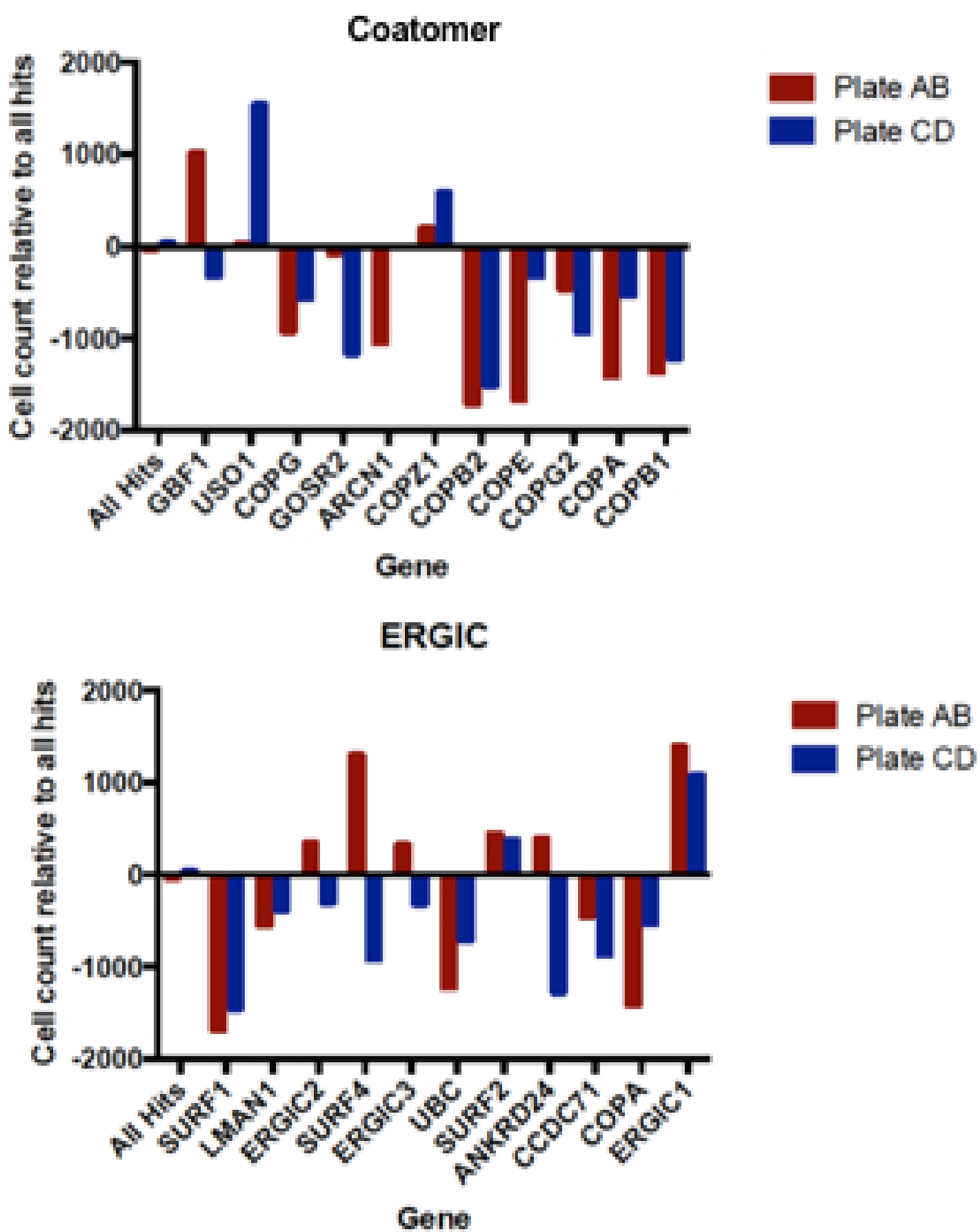

Supplement: S2 Fig — (TIF) [file pone.0166916.s002.tif]
